# Chinese Olive (*Canarium album* L.) Fruit Extract Attenuates Metabolic Dysfunction in Diabetic Rats

**DOI:** 10.3390/nu9101123

**Published:** 2017-10-15

**Authors:** Yu-Te Yeh, An-Na Chiang, Shu-Chen Hsieh

**Affiliations:** 1 Institute of Food Science and Technology, National Taiwan University, Taipei 106, Taiwan; d02641002@ntu.edu.tw; 2 Institute of Biochemistry and Molecular Biology, National Yang-Ming University, Taipei 112, Taiwan; anchia@ym.edu.tw

**Keywords:** Chinese olive fruit, high-fat diet, hyperglycemia, metabolic dysfunction, antioxidant activities, proinflammatory cytokines

## Abstract

Hyperglycemia and dysregulation of lipid metabolism play a crucial role in metabolic dysfunction. The aims of present study were to evaluate the ameliorative effect of the ethyl acetate fraction of Chinese olive fruit extract (CO-EtOAc) on high-fat diet (HFD) and streptozotocin (STZ)-induced diabetic rats. CO-EtOAc, rich in gallic acid and ellagic acid, could markedly decreased the body weight and epididymal adipose mass. In addition, CO-EtOAc increased serum HDL-C levels, hepatic GSH levels, and antioxidant enzyme activities; lowered blood glucose, serum levels of total cholesterol (TC), triglycerides (TG), bile acid, and tumor necrosis factor alpha (TNFα); and reduced TC and TG in liver. We further demonstrated that CO-EtOAc mildly suppressed hepatic levels of phosphorylated IRS-1, TNF-α, and IL-6, but enhanced Akt phosphorylation. The possible mechanisms of cholesterol metabolism were assessed by determining the expression of genes involved in cholesterol transportation, biosynthesis, and degradation. It was found that CO-EtOAc not only inhibited mRNA levels of *SREBP-2*, *HMG-CoAR*, *SR-B1*, and *CYP7A1* but also increased the expression of genes, such as *ABCA1* and *LDLR* that governed cholesterol efflux and cholesterol uptake. Moreover, the protein expressions of ABCA1 and LDLR were also significantly increased in the liver of rats supplemented with CO-EtOAc. We suggest that Chinese olive fruit may ameliorate metabolic dysfunction in diabetic rats under HFD challenge.

## 1. Introduction

Diabetes mellitus (DM) is one of the most common endocrine disorders and the third leading cause of death in developed countries [1]. Type 2 diabetes mellitus (T2DM) accounts for about 90% to 95% of all diagnosed cases of diabetes [2]. Accumulating evidence indicates that obesity contributes to down-regulation of insulin secretion, defecting insulin action or both [3,4], which may disturb carbohydrate and lipid homeostasis [5]. Moreover, T2DM is also associated with the chronic inflammatory state [6]. Until now, among traditional anti-diabetic drugs, metformin is the most used in clinical therapy, but the side effects such as lactic acidosis and permanent nerve damage, limit its application in certain populations [7]. Understanding the molecular mechanism underlying T2DM would definitely facilitate the development of treatment for T2DM [8].

Previous studies showed that obesity-induced diabetes results from defective insulin receptor-mediated signaling, which includes insulin receptor substrate 1 (IRS-1) and protein kinase B (Akt) [9], and is also associated with increased inflammatory response [10]. Dysregulation of inflammatory molecules and lipid profiles usually occurs in the insulin-resistant state of T2DM [11]. Additionally, oxidative stress also leads to insulin resistance and the pathophysiological outcomes of T2DM, such as β-cell dysfunction and impaired glucose tolerance [12]. Antioxidant enzymes possess the ability to scavenge endogenous free radicals and thus reduce the deleterious consequences that affect glucose and lipid metabolism in vivo [13]. Consistent to this idea, reduced glutathione (GSH) or anti-oxidant enzymes, such as superoxide dismutase (SOD), catalase (CAT), and glutathione peroxidase (GPx), exhibit the ability to ameliorate the pathophysiological problems of diabetes [14,15].

Extensive dysregulation of lipid metabolism and cholesterol homeostasis occurs in both obesity and T2DM [16]. Sterol regulatory element-binding protein-2 (SREBP-2) is a transcription factor involved in the regulation of endogenous cholesterol synthesis in the liver [17]. The hepatic enzyme 3-hydroxy-3-methyl-glutaryl-coenzyme A reductase (HMG-CoAR) is a downstream target of SREBP-2, responsible for the regulation of cholesterol synthesis. As for the clearance of cholesterol, low-density lipoprotein receptor (LDLR) plays a critical role in uptake of circulating LDL in peripheral tissues, whereas scavenger receptor class B type 1 (SR-B1) uptakes high-density lipoprotein cholesterol (HDL-C) from peripheral tissues to the liver via circulation [18]. Furthermore, adenosine triphosphate-binding cassette (ABC) transporters are abundantly expressed in multiple organs and provide a major function in the regulation of cholesterol homeostasis. ABCA1 is probably the most prominent member of the ABC superfamily and it is crucial for HDL-C formation and works as a cellular efflux transporter of cholesterol and lipids in the liver. Cholesterol can also be removed from hepatocytes by conversion to bile acids through the enzymes, cholesterol 7α-Hydroxylase (CYP7A1) and sterol 27-hydroxylase (CYP27A1). In the liver, ABCG5/G8 transporter is responsible for the efflux of hepatic cholesterol into the bile and subsequently increases the cholesterol metabolite secretion into feces. Thus, overexpression of ABCA1 or ABCG5/G8 in the liver can lower hepatic cholesterol [18].

Epidemiological studies suggest that polyphenols could offer strong protection against metabolic disorders through their anti-oxidative functions [19]. Chinese herbs contain a rich source of bioactive phytochemicals, especially polyphenols and flavonoids, which may provide antioxidant and anti-inflammatory functions as well as hypoglycemic and hypolipidemic potential [20]. It has been reported that Chinese olive (*Canarium album* L.) shows high phenolics content, strong antioxidant capacity and potent free radical-scavenging ability [21]. Chinese olive is a tropical and semi-tropical fruit of the family Burseraceae, is widely cultivated in Taiwan, the southeast China and other regions of Asia. Moreover, it is widely used for the treatment of faucitis, stomatitis, hepatitis and toxicosis [22]. Recent studies have shown that Chinese olive and its related phenolic compounds have a wide spectrum of clinical applications such as in anti-tumor growth, diabetes, and inflammation [21,23]. However, the molecular mechanisms of Chinese olive remain largely unknown. In the present study, we generated a rat model with the combination of high-fat diet (HFD) treatment and low-dose streptozotocin (STZ) injection. This animal model could mimic human type 2 diabetes mellitus that provides the phenotypes of hyperlipidemia, hyperglycemia and increased body weight [24]. Our aim was to evaluate whether treatment with ethyl acetate fraction of Chinese olive fruit extract (CO-EtOAc) had the potential to improve the metabolic abnormalities associated with diabetes under high fat and STZ challenge. Ellagic acid and gallic acid are the main compounds in CO-EtOAc, which may regulate lipid and glucose metabolic pathways. Our findings suggest that Chinese olive is a potential therapeutic herb for the treatment of metabolic disorders.

## 2. Materials and Methods

### 2.1. Preparation of Chinese Olive Fruit Extract

Chinese olive fruits were obtained from Baoshan Township, Hsinchu County, Taiwan. The olive fruit extract was prepared by the standard procedure as described [25]. In brief, olive fruit was extracted with 100% methanol and re-suspended with water in a ratio of 1:10 (*v*/*v*) and then partitioned with *N*-hexane, ethyl acetate, and *N*-butanol. The CO-EtOAc was stored at −20 °C until use and the same batch of Chinese olive fruit extract was used throughout this study. Composition of CO-EtOAc was determined using high-performance liquid chromatography (HPLC) analysis with a Photodiode Array Detector (PDA, Thermo Fisher Scientific, Waltham, MA, USA). Chromatographic separation was conducted using a 250 × 4.6 mm (i.d.), 5 μm, C-18 reversed-phase column (MACHEREY-NAGEL, Dueren, Germany). Gradient elution was performed with 0.5% (*v*/*v*) acetic acid (solvent A) and methanol (solvent B) at a constant flow rate of 0.6 mL min^−1^. The linear gradient profile was as followings: 94% A and 6% B at the start, 38% A and 62% B at 30 min, and 10% A and 90% B at 60 min. UV-Vis absorption spectra were recorded on-line at 200 and 600 nm during HPLC analysis.

### 2.2. Animals and Diets

Six-week-old male Sprague-Dawley rats (initial weights 130 ± 10 g) were obtained from BioLASCO, Taipei, Taiwan. The experimental protocol was approved by the Institutional Animal Care and Use Committee of the National Taiwan University, Taipei, Taiwan, Republic of China. Rats were randomly divided into four groups: (1) control group; (2) diabetic control (DC); (3) DC + CO-EtOAc (50 mg/kg body weight); and (4) DC + CO-EtOAc (150 mg/kg body weight). The rats besides the control group were fed with high-fat diet (HFD, 60% kcal fat), whereas rats of control group were fed with normal chow diet (12.6% kcal fat) during the whole experimental period. The animals were housed in a room at temperature of 23 ± 2 °C, relative humidity of 55 ± 5% and a 12 h light/dark cycle. In Week 2, HFD-fed rats were treated intragastrically with CO-EtOAc (dissolved in 0.5% carboxymethyl cellulose, 50 or 150 mg/kg/day), control animals were also gavaged with 0.5% carboxymethyl cellulose and this treatment continued daily for eight weeks (*n* = 10 in each group). To establish a diabetic animal model, the rats were treated with HFD combined with 35 mg/kg STZ (streptozotocin, Sigma-Aldrich, Inc., St. Louis, MO, USA), a compound that is particularly toxic to the insulin-producing beta cells following the modified method described by Srinivasan et al. [26]. In Week 7, experimental rats excluding control group were given a single intraperitoneal injection of STZ. All of the rats in DC group have been verified to be at hyperglycemic stage with fasting blood glucose levels higher than 300 mg/dL (16.7 mmol/L) after STZ administration for 72 h [27]. During the eight-week experimental period, rats were free to access food and water. Animals were sacrificed by CO_2_ anesthesia, and serum was collected for biochemical analysis. The liver was dissected and stored at −80 °C for further experiments.

### 2.3. Biochemical Analysis

Fasting blood samples were collected from the tail vein of the rats after starvation for 12 h. Fasting blood glucose (FBG) levels were determined by the glucose analyzer (Eumed Biotechnology Co., Ltd., Hsinchu, Taiwan). At the end of the experiment, serum samples were collected by cardiac puncture and centrifugation at 1500 g for 10 min. Levels of total cholesterol (TC), triglyceride (TG), HDL-cholesterol (HDL-C), aspartate aminotransferase (AST), and alanine aminotransferase (ALT) were measured by an automatic chemistry analyzer (SPOTCHEM EZ SP-4430, ARKRAY, Inc., Kyoto, Japan). Serum samples were applied into the strips for determination of HDL-C levels. After precipitation of LDL and VLDL fractions by reacting with the polyethylene glycol reagent, cholesterol ester of the HDL fraction was then catalyzed by cholesterol esterase to produce cholesterol and subsequently reacted with cholesterol oxidase and peroxidase to produce a final product of quinoneimine. HDL-C levels were then determined in the chemical analyzer. Insulin levels were determined using commercial kits (Mercodia AB, Uppsala, Sweden). Serum bile acid and TNF-α levels were determined using kits according to the manufacturer’s instructions (Cayman, Ann Arbor, MI, USA).

### 2.4. Quantification of Hepatic Triglyceride and Cholesterol Levels

Lipids were extracted from the liver tissue (1.5 g) according to the method described by Folch et al. [28]. Briefly, total lipids were extracted from the liver samples by homogenizing the tissues using a mixed 8:4:3 chloroform/methanol/0.9% NaCl (*v*/*v*/*v*) to a final dilution of 20-fold original volume of the tissue sample. The organic layer was then separated, evaporated, and reconstituted in chloroform. The values of TG and TC were measured using a colorimetric assay kit (Sigma-Aldrich, Los Angeles, CA, USA) and the results were expressed as mg per gram of the liver weight.

### 2.5. Measurement of Hepatic Antioxidant Status and TBARS Levels

Hepatic levels of reduced glutathione (GSH) and activities of superoxide dismutase (SOD), catalase (CAT), and glutathione peroxidase (GPx) were determined in the extract of liver homogenates using commercial kits ((Cayman, Ann Arbor, MI, USA). Briefly, SOD activities were measured by detecting the reduction of a tetrazolium salt at 450 nm, which was a superoxide radical generated by xanthine oxidase and hypoxanthine. One unit of SOD is defined as the amount of enzyme needed to exhibit 50% dismutation of the superoxide radical. This assay measures all three types of SOD (Cu/Zn, Mn, and FeSOD). Glutathione peroxidase (GPx) activity was quantified by a coupled assay with glutathione reductase (GR)-catalyzed oxidation of NADPH. The oxidized glutathione (GSSG) formed after reduction of hydroperoxide by GPx is recycled to its reduced state by GR in the presence of NADPH. Measurements were made at 340 nm and expressed in µmol/min per mg protein. The GSH level was assessed using the protocol for the glutathione assay kit, which contains GSH reductase, 5,5′-dithio-bis-2-nitrobenzoic acid and Ellman’s reagent. The 5-thio-2-nitrobenzoic acid (TNB) generated after reaction between the sulfhydryl group of GSH and 5,5′-dithio-bis-2-nitrobenzoic acid can be measured at 405 nm using a microplate reader. Catalase activity was detected in the presence of hydrogen peroxide. The formaldehyde produced was measured with 4-amino-3-hydrazino-5-mercapto-1,2,4-triazole and the absorbance was monitored at 540 nm. The level of lipid peroxidation was assayed by determining the production of thiobarbituric acid reactive substances (TBARS). In brief, 0.5 g liver tissues were homogenized in 1% KCl (*w*/*v*). The homogenized solution was mixed with the TBA reagents (20% trichloroacetic acid and 1% butylated hydroxytoluene and incubated at 90 °C for 20 min and then stopped the reaction on ice. After cooling to room temperature, a mixture of solvents *N*-butanol and pyridine (15:1; *v*/*v*) was added. The mixture was mixed thoroughly and separated by centrifugation at 3000 g for 5 min. The organic layer was taken out and TBARS level was measured at 535 nm using a standard curve of thiobarbituric acid (TBA) adduct formation with freshly diluted 1,1,3,3-tetraethoxypropane.

### 2.6. Histological Analysis

The right medial lobes of rat liver tissues were fixed in 10% neutral formalin, embedded in 100% paraffin, and cut to 4–5 µm-thick sections. Each section was deparaffinized by incubating in xylene, hydrated in a descending series of ethanol (100%, 95%, 80%, and 70%), and washed with distilled water. Afterwards, each section was rapidly dehydrated in an increasing series of ethanol (70%, 95%, and 100%). Finally, the sections were treated with xylene and mounted on glass slides, stained with hematoxylin and eosin (H&E) and examined by a light microscope under 200× magnification. The score counting of histological analyses were following the criteria described by Garcimartin et al. [29]. Interpretation of the scores was following the definition provided by Kleiner et al. [30].

### 2.7. Quantitative Reverse Transcription Polymerase Chain Reaction (RT-qPCR)

Total RNA was extracted using TRIZOL reagent (Invitrogen Corp., Carlsbad, CA, USA), following the manufacturer’s instructions. The purified RNA (2 μg) was treated with RNase-free DNase I (Invitrogen), and then reverse-transcribed with oligo (dT) primer using the SuperScript First-Strand Synthesis System (Invitrogen) to generate cDNA. The qPCR reaction mixture consisted of 10 μL total volume solution containing 5 μL of 2X KAPA SYBR^®^FAST qPCR Master Mix ABI Prism™, 0.2 μL of 10 μM of each primer, 10 ng cDNA template and distilled water. The reactions were carried out on a StepOnePlus™ Real-Time PCR Systems (Thermo Scientific, Waltham, MA, USA). The primer sequence of each gene is shown in Table 1. The relative copy number was calculated using the threshold crossing point (Ct) as calculated using the 2^−ΔΔCt^ method [31] and the relative gene expression was normalized to the amount of GAPDH rRNA.

### 2.8. Western Blotting

Tissue cells were lysed in lysis buffer (20 mM Tris-HCl, pH 7.5, 150 mM NaCl, 1 mM EDTA, 1 mM EGTA, 1% Triton X-100, 50 mM dithiothreitol, complete protease inhibitor cocktail (Roche Diagnostics GmbH, Mannheim, Germany) and phosphatase inhibitor cocktail I and II (Sigma-Aldrich, Inc., St. Louis, MO, USA)). Cell lysates were centrifuged and the supernatants were collected. The protein concentration was determined using Bradford reagent (Bio-Rad, Hercules, CA, USA) with BSA as the standard. Equal amounts of protein were separated by SDS-PAGE and transferred to nitrocellulose membranes after gel electrophoresis. The blots were blocked with 5% (*w*/*v*) skim milk and probed with antibodies against CPT-1 (Abcam, Cambridge, MA, USA), AMPKα, phospho-AMPKα (Thr-172), LDLR, ABCA1 (Gene Tex, Irvine, CA, USA), IRS-1, phospho-IRS-1 (Ser-307), Akt, and phospho-Akt (Ser-473) (Cell Signaling Technology, Beverly, MA, USA) separately, followed by goat anti-rabbit or mouse IgG horseradish peroxidase (HRP)-conjugated secondary antibodies. The protein bands were visualized using enhanced chemiluminescence reagents (ECL, PerkinElmer, Boston, MA, USA). The intensity of each band was quantified by densitometry using Image Quant software (Molecular Dynamics, Sunnyvale, CA, USA). The blots were then stripped for further probing with β-actin or α-tubulin antibodies (Cell Signaling Technology, Beverly, MA, USA) as an internal control.

### 2.9. Statistical Analysis

All data are expressed as mean ± SEM of at least three independent experiments. Significant differences were analyzed by one-way ANOVA, followed by Duncan’s multiple range tests using SAS 9.0 software (Cary, NC, USA). *p* < 0.05 was considered statistically significant between means of two groups. The Mann–Whitney U test, evaluated by the Monte Carlo method for small samples, was used to evaluate NAFLD histological data. Pearson correlations between selected variables were tested.

## 3. Results

### 3.1. High Performance Liquid Chromatography (HPLC) Analysis of CO-EtOAc

**Figure 1:**
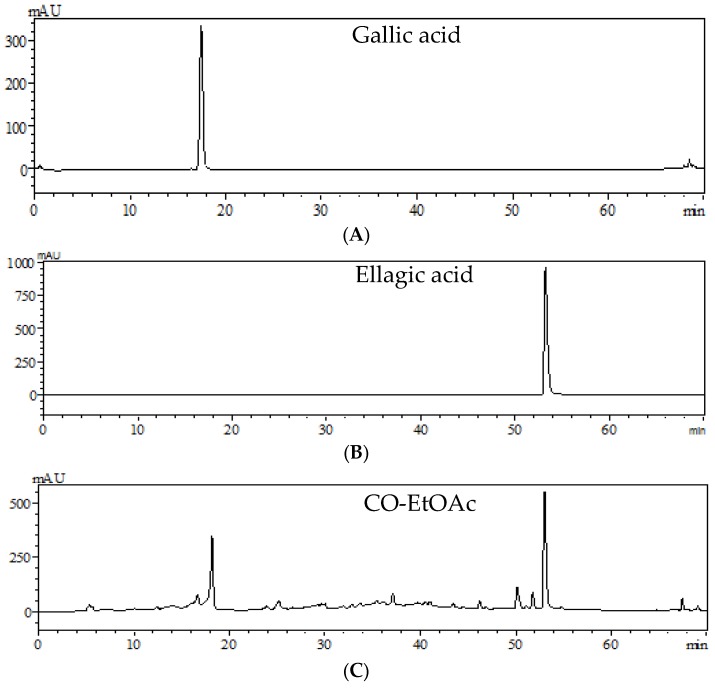
High performance liquid chromatography (HPLC) pattern of polyphenol standards and CO-EtOAc: (**A**) HPLC chromatogram of standard reference: gallic acid (GA, 17.31 min); (**B**) standard reference: ellagic acid (EA, 53.22 min); and (**C**) HPLC chromatogram of CO-EtOAc.

The major compounds of CO-EtOAc were analyzed by HPLC. As shown in Figure 1C, two major peaks indicated gallic acid and ellagic acid, respectively, as compared to standard references of gallic acid appeared at 17.31 (Figure 1A) and ellagic acid at 53.22 min (Figure 1B). Moreover, the contents (EA and gallic acid) of CO-EtOAc were 7.8 and 11.2 mg/g extract, respectively.

### 3.2. The Effect of CO-EtOAc on Body Weight, Food Intake and Biochemical Parameters

All of the rats used in this study remained healthy in appearance to the end of the experiment. As shown in Table 2, rats fed with HFD combined with STZ injection (DC group) had a significant increase in body weight, but daily food intake did not significantly differ among groups. Epididymal adipose tissue (EAT) weight and blood glucose levels were significantly increased in DC group. However, administration of 150 mg/kg CO-EtOAc significantly decreased the gain of body weights by 12.9%, the gain of the epididymal adipose tissue weights by 33.4%, and the increase in blood glucose levels by 202% when compared with DC group. The levels of serum TC, TG, bile acid, TNF-α, AST and ALT were significantly increased in the diabetic rats, whereas these biochemical parameters were suppressed to near those in the control rats after the higher dose of CO-EtOAc administration. Moreover, we found that the serum HDL-C levels were significantly increased in rats treated with 50 and 150 mg/kg CO-EtOAc by 27.2% and 53.1%, respectively, when compared with the rats in the DC group.

### 3.3. The Effect of CO-EtOAc on Hepatic Antioxidant Enzyme Activities and TBARS Levels

To evaluate the effect of CO-EtOAc on redox status, we determined the hepatic antioxidant enzyme activities in rats treated with CO-EtOAc (Table 3). HFD combined with STZ resulted in a significant decrease in hepatic GSH levels and activities of SOD, GPx, and CAT, whereas treatment with 150 mg/kg CO-EtOAc reversed the suppression of these antioxidant enzyme activities. This finding reveals that CO-EtOAc significantly restored the HFD and STZ-mediated suppression of antioxidant enzyme activities. On the other hand, we also evaluated the effect of CO-EtOAc on lipid peroxidation, which was estimated by measuring the levels of thiobarbituric acid reactive substances (TBARS). As shown in Table 3, HFD combined with STZ significantly enhanced the TBARS values by 2.8-fold, whereas CO-EtOAc inhibited the induction. These results suggest that CO-EtOAc might reverse the HFD-induced oxidative damage in the liver through the increase in antioxidant enzyme activities and decrease in lipid peroxidation.

### 3.4. The Effect of CO-EtOAc on the Regulation of Insulin Signaling

**Figure 2:**
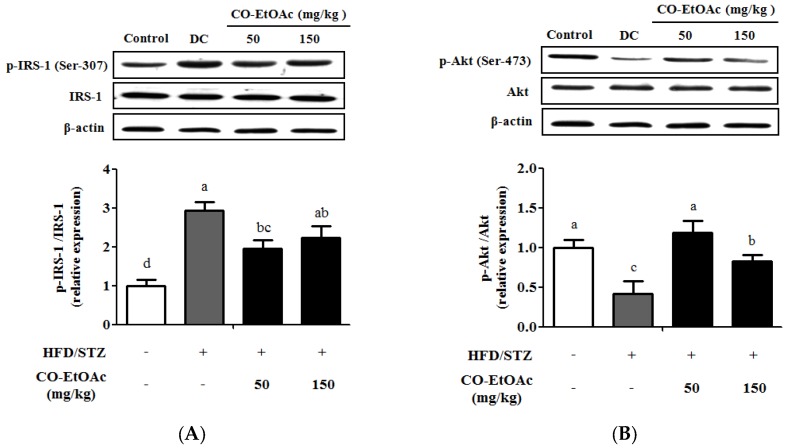
Effects of CO-EtOAc on the regulation of insulin receptor-mediated signaling. Relative protein levels of hepatic: (**A**) IRS-1 (phosphorylated IRS-1 at Ser-307 and total IRS-1); and (**B**) Akt (phosphorylated Akt at Ser-473 and total Akt) were detected by Western blot analysis. Data are represented as mean ± SEM (*n* = 8–10). Values with different letters are statistically different (*p* < 0.05) by analysis of variance (ANOVA) followed by Duncan’s multiple range tests. Relative expression values are expressed as folds of the control group, which is set as 1.

Considering that hyperglycemia apparently occurred in the DC group, we examined whether CO-EtOAc plays a role in the regulation of insulin signaling. Insulin receptor-mediated signaling transduction links the metabolic pathway of insulin actions with the Akt pathway [32]. As shown in Figure 2A, DC rats showed a significant increase in phosphorylated IRS-1 (at Ser-307), which was decreased upon both 50 mg/kg and 150 mg/kg CO-EtOAc treatment. Moreover, 50 and 150 mg/kg CO-EtOAc up-regulated phosphorylated Akt protein expression by 2.4-fold and 1.7-fold compared to the DC group, respectively (Figure 2B).

### 3.5. The Effect of CO-EtOAc on Hepatic Lipid Accumulation

**Figure 3:**
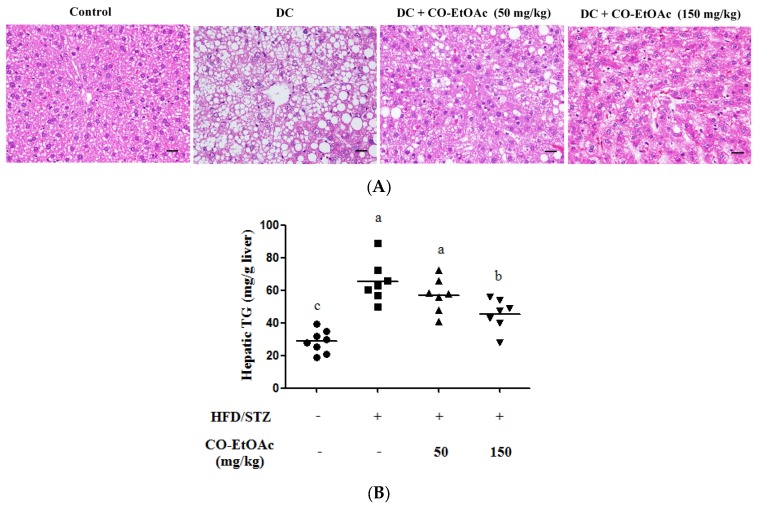
Effects of OE-EtOAc on hepatic lipid accumulation. (**A**) The sections of rat liver tissues were stained by H&E with a representative photograph under 200× magnification; scale bar represents 30 μm. (**B**) The triglyceride content was analyzed using enzymatic assay. Data are represented as mean ± SEM (*n* = 8–10). Values with different letters indicate statistical difference (*p* < 0.05) by analysis of variance (ANOVA) followed by Duncan’s multiple range tests.

Results from the histological analysis of liver tissue showed that steatosis was occurred in the liver of rats in DC group. Hepatocyte ballooning was clearly found in DC rats, whereas these effects were reversed in the CO-EtOAc-treated rats (Figure 3A). Rats in the DC group developed full spectrum of steatohepatitis, including steatosis, severe inflammation, and hepatocellular ballooning, resulting in a mean non-alcoholic fatty liver disease activity score (NAS) of 5.6, significantly higher than the value in control rats (NAS = 1.9) (*p* < 0.05) (Figure 3A and Table S1). However, treatment with CO-EtOAc significantly improved the phenomenon of steatohepatitis. Furthermore, hepatic triglyceride levels in DC group were increased by 127.4% (vs. control group). Administration of 150 mg/kg CO-EtOAc attenuated the increase of triglyceride levels by 56.8% when compared to the DC group (Figure 3B). These findings indicate that CO-EtOAc might have protective effects against high fat diet-induced lipid accumulation in the liver.

### 3.6. The Effect of CO-EtOAc on Hepatic Cholesterol levels and Gene Expression

**Figure 4:**
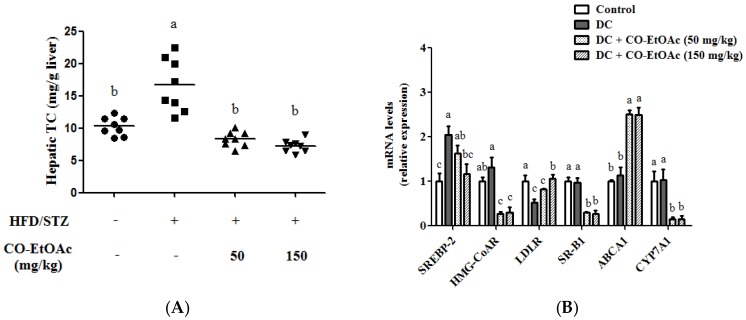

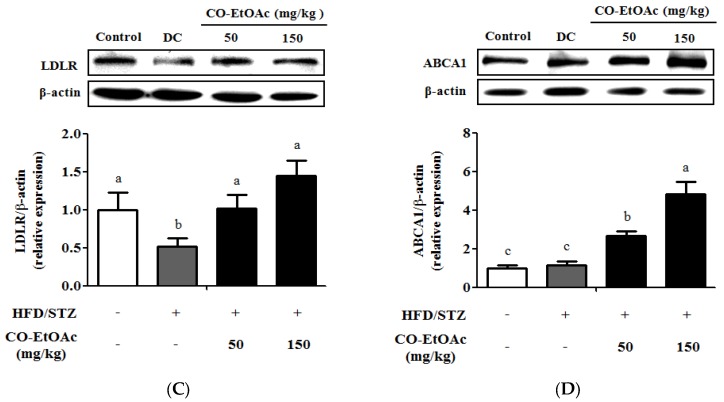
Effects of OE-EtOAc on the levels of hepatic cholesterol and gene expression. (**A**) The content of hepatic cholesterol was analyzed using enzymatic assay. (**B**) Relative mRNA levels of hepatic *SREBP-2*, *HMG-CoAR*, *LDLR*, *SR-B1*, *ABCA1*, and *CYP7A1* were analyzed using RT-qPCR. (**C**) Relative protein levels of hepatic LDLR and ABCA1 were detected by Western blot analysis. Data are represented as mean ± SEM (*n* = 8–10). Values with different letters show significant difference (*p* < 0.05) by analysis of variance (ANOVA) followed by Duncan’s multiple range tests. Quantitative values of mRNA and protein expression are expressed as folds of the control group, which is set as 1.

Hepatic cholesterol levels were remarkably increased in the DC group by 61.8%. However, treatment with 150 mg/kg CO-EtOAc blocked the increase (Figure 4A). Furthermore, we assessed whether CO-EtOAc was able to affect the expression of genes that govern cholesterol synthesis, transportation and degradation. As shown in Figure 4B, CO-EtOAc apparently suppressed the mRNA levels of *SREBP-2*, *HMG-CoAR*, *SR-B1*, and *CYP7A1*, but increased *LDLR* and *ABCA1* mRNA levels. Consistently, protein levels of LDLR and ABCA1 were significantly increased by the treatment of CO-EtOAc (Figure 4C,D, respectively). These results show that CO-EtOAc exhibits a broad range of actions in the regulation of cholesterol synthesis and transportation in animal livers.

### 3.7. The Effect of CO-EtOAc on Expression of Inflammatory Cytokines

**Figure 5:**
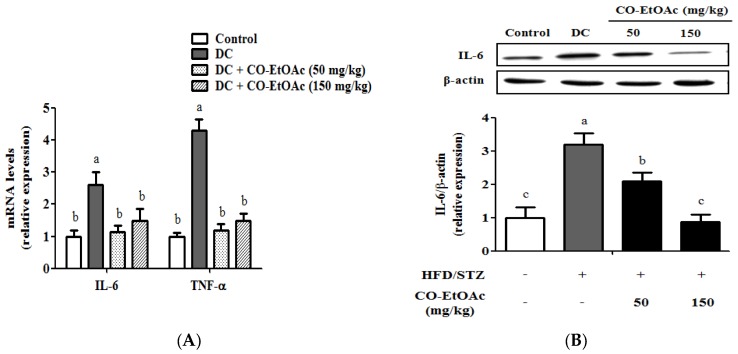

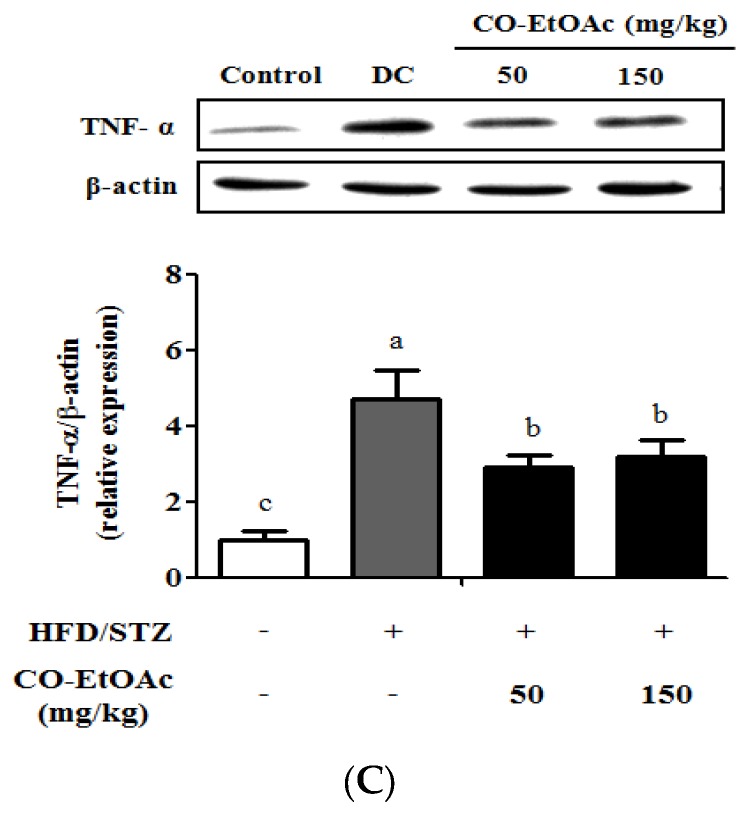
Effects of OE-EtOAc on the expression of hepatic inflammatory cytokines. (**A**) Relative mRNA levels of hepatic IL-6 and TNF-α were determined using RT-qPCR. Relative protein levels of: hepatic IL-6 (**B**); and TNF-α (**C**) were examined by Western blot analysis. Data are represented as mean ± SEM (*n* = 8–10). Values with different letters are significantly different (*p* < 0.05) by analysis of variance (ANOVA) followed by Duncan’s multiple range tests. Quantitative values of mRNA and protein expression are expressed as folds of the control group, which is set as 1.

Overproduction of inflammatory cytokines is usually a risk factor for metabolic diseases [33]. We thus investigated whether CO-EtOAc administration could modulate the hepatic inflammatory cytokines. As shown in Figure 5A, IL-6 and TNF-α mRNA levels were highly elevated in the DC group. Consistently, the protein levels of IL-6 and TNF-α were significantly increased in the DC group as compared to the control group by 3.1-fold (*p* < 0.01) and 4.2-fold (*p* < 0.01), respectively (Figure 5B). However, administration of CO-EtOAc significantly reduced the IL-6 and TNF-α mRNA and protein levels. The results showed that CO-EtOAc may attenuate hepatic inflammatory stress in the HFD-fed diabetic animals.

## 4. Discussion

A number of phenolic compounds with anti-hyperlipidemia or anti-hyperglycemia potential have been identified in Chinese olive fruit. Our previous study identified gallic acid and ellagic acid as the major compounds in CO-EtOAc [34]. It has been reported that gallic acid not only improves glucose tolerance, triglyceride concentration, total cholesterol, and LDL-cholesterol in diet-induced obesity animals [35,36] but also attenuates high-fat diet fed-streptozotocin-induced insulin resistance via partial agonism of peroxisome proliferator-activated receptor gamma (PPARγ) in experimental type 2 diabetic rats and enhances glucose uptake through translocation and activation of glucose transporter 4 in PI3K/p-Akt signaling pathway [37]. The other phenolic compound, ellagic acid, has been shown to regulate plasma glucose in STZ-induced diabetic rats [38], attenuate high-carbohydrate, high-fat diet-induced metabolic syndrome in rats [39], decrease oxidized LDL uptake and stimulate cholesterol efflux in murine macrophages [40]. Through AMPK activation, ellagic acid stimulates glucose transport in adipocytes and muscles [41], and it downregulates macrophage lipid uptake to block foam cell formation of macrophages and boost cholesterol efflux in lipid-laden foam cells [40].

Excessive intake of energy raises fat accumulation in adipose tissue and liver, which leads to impaired lipid metabolism, inflammatory signaling, and insulin sensitivity [42,43]. It has been reported that HFD induces obesity and insulin resistance, whereas low doses of intraperitoneal STZ induce mild impairment of insulin secretion, which is similar to the feature in the late stage of T2DM [44,45]. Therefore, a T2DM rat model has been established by a standardized HFD treatment and low-dose STZ challenge in rats. Currently, this rat model is widely used to develop therapeutics in diabetes studies [46,47]. Epidemiological studies strongly suggest that intakes of phytochemical-rich foods have beneficial effects on lipid and glucose metabolism as well as the amelioration of inflammation [48]. In addition, medicinal plants are frequently considered to be less toxic and free from side effects than synthetic or clinical drugs [49]. However, the effect of Chinese olive extracts on HFD-mediated metabolic disorder in the liver is still unknown. Herein, we evaluated the role of CO-EtOAc in hepatic lipid accumulation and regulation of genes and proteins involved in lipid and carbohydrate metabolism in HFD-fed rats combined with STZ challenge.

Various evidence has demonstrated the association among obesity, chronic inflammation, and T2MD [50,51]. Two essential inflammatory cytokines, TNF-α and IL-6, affect adipose tissue and the liver, and are involved in the metabolic complications of T2MD [52,53]. Macrophages of adipose tissue are the principal source of pro-inflammatory cytokines and chemokines production. Once macrophages are activated, the inflammatory signaling is subsequently propagated and then insulin sensitivity is interfered in the insulin target tissues to develop IR [54]. Prolonged IR leads to decreased pancreatic function, and increased gluconeogenesis, which increase fasting glucose level, as observed in T2MD patients [55]. Interestingly, HFD-induced obesity causes inflammation in the adipose tissue prior to the liver [56]. In the histological studies (Table S1), we found that both hepatic steatosis and hepatocellular ballooning were not significantly improved upon 50 mg/kg CO-EtOA treatment, while 150 mg/kg CO-EtOAc provided considerable improvement. Decreases in the hepatic levels of TG and serum levels of TG, ALT and AST were coordinate with the improvement of hepatic steatosis caused by HFD. In addition, both severe lobular and portal inflammation were attenuated in rats treated with CO-EtOAc in a dose dependent manner, which is consistent with the improvement in levels of inflammatory cytokines and anti-oxidative capacity.

It has been reported that TNF-α and IL-6 can increase IRS serine/threonine phosphorylation, which negatively modulates insulin signaling transduction and ultimately decreases insulin sensitivity [11,57]. IRS-1 is a cytoplasmic substrate for the insulin receptor and it plays a major role in facilitating glucose uptake and glycogen synthesis [58,59]. The previous report showed that the STZ-induced diabetic rats had increased hepatic p-IRS-1 (Ser^307^) level, but decreased p-Akt (Ser^473^) level [60]. Consistently, elevated p-IRS-1 (Ser^307^) and reduced p-Akt (Ser^473^) levels were found in the livers of diabetic rats in the present study, and these changes could be significantly ameliorated by CO-EtOAc treatment. However, in contrast to the dose-dependent inhibitory effects of CO-EtOAc on hepatic steatosis, it seems that higher dose of CO-EtOAc did not provide stronger effects on reverting insulin signaling, and the similar phenomenon can also be observed in the blood glucose lowering effects of CO-EtOAc. We propose different compounds within CO-EtOAc might separately regulate insulin signaling and lipid metabolism. It is also possible that different pathways respond to same compounds with different sensitivity.

The major compound GA of CO-EtOAc has been reported to up-regulate the expression of proteins related to insulin signal transduction, including insulin receptor, insulin receptor substrate 1, phosphatidylinositol-3 kinase, Akt/protein kinase B, and glucose transporter 2 [61]. Combined with the results of decreased circulating levels of TNF-α and IL-6, we suggest that CO-EtOAc might be able to improve insulin sensitivity via regulation of inflammatory signaling.

Several studies have shown that hyperlipidemia leads to liver injury and insulin resistance through oxidative stress [62,63]. However, oxidative damage can be attenuated by the enzymatic and non-enzymatic antioxidant defense systems in various tissues. GSH and antioxidant enzymes, such as SOD, CAT, and GPx can scavenge free radicals and reduce oxidative stress in tissues [64]. GSH plays as the first line defense against free radicals in the liver and is also responsible for the maintenance of protein thiols and acts as a substrate for GPx and GST [65]. GSH content has been reported to be depleted in obese rats fed with HFD and the depletion can be restored after the treatment of GA [66]. In this study, we show that CO-EtOAc administration increased GSH levels and activities of SOD, CAT, and GPx in the livers of diabetic rats. Moreover, treatment with CO-EtOAc alleviated the increase in MDA levels in diabetic rats, implying a reduction in hepatic lipid peroxidation. Furthermore, serum levels of AST and ALT were also reduced in the CO-EtOAc-treated rats. We thus propose that CO-EtOAc can protect diabetic rats from liver damage via activation of endogenous antioxidant enzyme activities.

Experiments and clinical cases indicate that T2DM is commonly accompanied by low levels of HDL-C and high levels of LDL-C, which result from cholesterol dysmetabolism [34]. Cholesterol homeostasis is maintained by three different pathways in the liver: de novo cholesterol synthesis, cholesterol uptake, and cholesterol metabolite (bile acids) secretion [67]. In mammals, SREBP-2 is mainly responsible for the regulation of HMG-CoA reductase (HMG-CoAR), a rate-limiting enzyme in de novo cholesterol synthesis, which can be elevated by high-fat diet feeding [68]. Raz et al. reported that inhibitors of hepatic HMG-CoAR are commonly used as drugs for the treatment of hypercholesterolemia and decrease the incidence of dyslipidemia in diabetic subjects [69]. The contribution of de novo cholesterol synthesis versus dietary intake for total body cholesterol has been estimated to be at a ratio of 70:30 [70]. In the present study, the mechanism behind the modulation of CO-EtOAc on reduction of serum and hepatic total cholesterol possibly result from decreasing gene expression of SREBP-2 and HMG-CoAR that regulate de novo cholesterol biosynthesis.

Hepatic nascent HDL-C formation is highly dependent on ABCA1 expression [71], which mediates the efflux of cholesterol and phospholipids to lipid-poor apolipoproteins (apo-A1 and apoE) [72]. The expression of the ABCA1 protein under diabetic states is controversial. A previous study reported that high-fat/high-cholesterol diet results in elevation of hepatic ABCA1 levels and plasma HDL-C levels [73]. However, other studies show decreased ABCA1 mRNA expression in the livers of STZ-induced diabetic mice [74,75]. The differences in regulation of ABCA1 expression under diabetic conditions might be attributed to the animal models used, type of diabetes studied, and its duration. Park et al. reported that ellagic acid increased cholesterol efflux from lipid-loaded macrophages by induction of ABCA1 expression and then transferred cholesterol onto lipid-poor apolipoproteins to form HDL particles [40]. In our study, CO-EtOAc significantly increased ABCA1 and serum HDL-C levels in rats under HFD treatment and STZ challenge, although no significant change was observed in diabetic rats when compared with control rats. Furthermore, biliary secretion of cholesterol, either in the form of free cholesterol or bile acids, is the route for eliminating cholesterol from body in mammals [76]. CYP7A1, the rate-limiting enzyme in bile acid synthesis, could be increased in the liver of animals consuming a high-fat diet combined with STZ administration [77]. Our data showed that hepatic CYP7A1 was significantly increased in diabetic rats, whereas CO-EtOAc decreased CYP7A1 gene expression and circulation of bile acid, which is probably due to the significant reduction of endogenous cholesterol synthesis caused by CO-EtOAc. The results of this study infer that CO-EtOAc could change the metabolism of hepatic cholesterol biosynthesis, transportation, and degradation.

## 5. Conclusions

**Figure 6:**
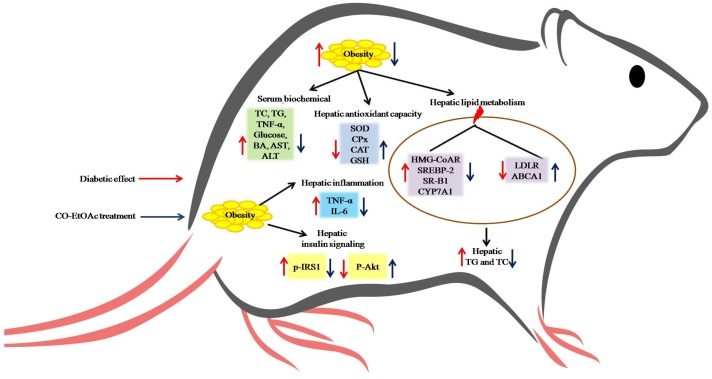
The potential mechanisms of CO-EtOAc protect metabolic dysfunction.

This study demonstrated that treatment of CO-EtOAc decreased body weight gain and altered serum lipid and inflammatory profiles in HFD-fed combined with STZ-challenged diabetic rats. We claimed the ameliorative effects of CO-EtOAc on hepatic lipid accumulation and glucose homeostasis, which might be regulated through PI3K/AKT pathway. Furthermore, CO-EtOAc suppressed hepatic gene and protein levels of IL-6 and TNF-α, but enhanced antioxidant enzyme activities. We addressed the molecular mechanisms of glucose and lipid metabolism affected by CO-EtOAc through examining the expression of phosphorylated-IRS-1 and Akt, *SREBP-2*, *HMG-CoAR*, *SR-B1*, and *CYP7A1* (Figure 6). These findings provide insights into the therapeutic potential of Chinese olive fruit extract in the management of metabolic disorders in diabetic animals.

## Figures and Tables

**Table 1:** Primers used for Quantitative Reverse Transcription Polymerase Chain Reaction (RT-qPCR).

Genes	Sequence of Forward and Reverse Primers (5′ to 3′)	Annealing Temperature (°C)	Accession No.
SREBP-2	FP: AGACTTGGTCATGGGGACAG RP:GGGGAGACATCAGAAGGACA	60 °C	NM_001033694
HMG-CoAR	FP: CCCAGCCTACAAACTGGAAA RP:CCATTGGCACCTGGTACTCT	55 °C	NM_013134
LDLR	FP: CAGCTCTGTGTGAACCTGGA RP:TTCTTCAGGTTGGGGATCAG	55 °C	NM_175762
SR-B1	FP: TGCCCCAGGTTCTTCACTAC RP:CCCTACAGCTTGGCTTCTTG	60 °C	NM_031541
ABCA1	FP: GTACCCAGCGTCCTTTGTGT RP:CCCAAGAGAGTGGAGAGACG	58 °C	NM_178095
ABCG1	FP: CTGCAAGAGAGGGATGAAGG RP:ACAGGAGGGTTGTTGACCAG	58 °C	NM_178095
CYP7A1	FP: CACCATTCCTGCAACCTTTT RP:GTACCGGCAGGTCATTCAGT	60 °C	NM_012942
TNF-α	FP: AAATGGGCTCCCTCTCATCAG RP:TTCTCTGCTTGGTGGTTTGCTACGAC	58 °C	NM_012675
IL-6	FP: TCTCTCCGCAAGAGACTTCCA RP:ATACTGGTCTGTTGTGGGTGG	60 °C	NM_012589.2
GAPDH	FP: AGACAGCCGCATCTTCTTGT RP:CTTGCCGTGGGTAGAGTCAT	60 °C	NM_017008

SREBP-2, sterol regulatory element-binding protein 2; HMG-CoAR, hydroxyl-3-methylglutaryl coenzyme A reductase; LDLR, low density lipoprotein receptor; SR-B1, scavenger receptor class B type I; ABCA1, ATP-binding cassette transporter A1; ABCG1, ATP binding cassette transporter G1; CYP7A1, cholesterol 7α-hydroxylase1; TNF-α, tumor necrosis factor alpha; IL-6, Interleukin 6; GAPDH, glyceraldehyde 3-phosphate dehydrogenase.

**Table 2:** Body weight, food intake and biochemical characteristics in diabetic rats.

Measurements	Control	DC	DC + CO-EtOAc (50 mg/kg)	DC + CO-EtOAc (150 mg/kg)
Body weight				
Wk-0 BW (g)	153.42 ± 6.52 ^a^	151.87 ± 4.67 ^a^	159.01 ± 3.55 ^a^	154.33 ± 5.64 ^a^
Wk-2 BW (g)	271.13 ± 9.33 ^b^	308.72 ± 10.33 ^a^	309.69 ± 11.65 ^a^	297.45 ± 7.65 ^a^
Wk-8 BW (g)	414.43 ± 9.74 ^b^	448.62 ± 15.68 ^a^	440.11 ± 23.19 ^a^	411.55 ± 13.95 ^b^
Body weight gain (g)	143.30 ± 0.41 ^b^	139.90 ± 5.35 ^a^	130.42 ±11.54 ^a^	114.10 ± 6.30 ^c^
Food intake (g/d)	27.61 ± 2.05 ^a^	25.64 ± 3.72 ^a^	24.76 ± 4.63 ^a^	25.76 ± 3.68 ^a^
EAT weight (g)	4.51 ± 0.27 ^c^	7.54 ± 1.71 ^a^	5.92 ± 1.33 ^b^	5.02 ± 0.22 ^b^
BG (mg/dL)	102.75 ± 7.95 ^d^	309.3 ± 10.56 ^a^	174.42 ± 10.56 ^c^	252.55 ±12.10 ^b^
Serum				
Insulin (ng/mL)	1.25 ± 0.24 ^b^	0.60 ± 0.18 ^a^	0.55 ± 0.19 ^a^	0.53 ± 0.12 ^a^
TC (mg/dL)	50.43 ± 8.50 ^b^	71.04 ± 7.01 ^a^	49.53 ± 12.60 ^b^	42.88 ± 10.32 ^b^
HDL-C (mg/dL)	27.38 ± 0.91 ^b^	22.13 ± 1.16 ^b^	28.13 ± 0.81 ^a^	33.88 ± 2.61 ^a^
BA (mg/dL)	30.53 ± 4.93 ^c^	50.48 ± 6.55 ^a^	41.25 ± 5.85 ^b^	35.75 ± 7.22 ^b,c^
TG (mg/dL)	75.25 ± 7.51 ^c^	145.42 ± 18.61 ^b^	92.45 ± 10.14 ^a^	72.22 ± 9.22 ^a^
TNF-α (pg/mL)	4.52 ± 0.25 ^c^	7.83 ± 1.66 ^a^	5.27 ± 0.48 ^b^	4.84 ± 0.80 ^c^
AST (IU/L)	38.57 ± 4.41 ^b^	71.30 ± 16.42 ^a^	40.83 ± 9.46 ^b^	33.14 ± 7.41 ^b^
ALT (IU/L)	20.57 ± 7.88 ^b^	47.30 ± 12.30 ^a^	25.83 ± 6.46 ^b^	23.14 ± 4.35 ^b^

1 BW, body weight; BG, blood glucose; BA, bile acid; DC, diabetic control; CO-EtOAc, ethyl acetate fraction of Chinese olive; EAT, epididymal adipose tissue; TC, total cholesterol; TG, triacylglycerol; TNF-α, Tumor necrosis factor alpha; HDL-C, High-density lipoprotein; AST, aspartate aminotransferase; ALT alanine aminotransferase. 2 Data are expressed as the mean ± SEM (*n* = 8–10). Values with different letters are significantly different (*p* < 0.05) by using one-way ANOVA coupled with Duncan’s multiple range tests. 3 Details of the nutrient contents, feeding, and treatment period are given in the “Materials and Methods” section.

**Table 3:** Antioxidant status and TBARS levels of the liver in diabetic rats.

Measurements	Control	DC	DC + CO-EtOAc (50 mg/kg)	DC + CO-EtOAc (150 mg/kg)
SOD (U mg protein^−1^)	83.33 ± 8.88 ^a^	59.37 ± 11.96 ^b^	65.37 ± 12.45 ^ab^	85.37 ± 10.23 ^a^
GSH (μmol mg protein^−1^)	32.55 ± 3.72 ^a^	17.72 ± 2.86 ^b^	28.64 ± 4.41 ^ab^	36.72 ± 4.86 ^a^
GPx (nmol mg protein^−1^)	101.51 ± 10.37 ^a^	60.5 ± 8.69 ^b^	76.5 ± 6.43 ^ab^	92.5 ± 4.69 ^a^
CAT (U mg protein^−1^)	59.32 ± 6.52 ^a^	21.5 ± 4.43 ^cd^	28.5 ± 2.43 ^c^	50.5 ± 4.38 ^b^
TBARS (nmol mg protein^−1^)	1.13 ± 0.71 ^c^	3.12 ± 0.73 ^a^	1.82 ± 0.35 ^b^	1.54 ± 0.27 ^b^

1 DC, diabetic control; CO-EtOAc, ethyl acetate fraction of Chinese olive; SOD, superoxidase dismutase; GSH, reduced glutathione; GPx, glutathione peroxidase; CAT, catalase; TBARS, 2-thiobarbituric acid reactive substances. 2 Data are expressed as the mean ± SEM (*n* = 8–10). Values with different letters are significantly different (*p* < 0.05) by using one-way ANOVA coupled with Duncan’s multiple range tests.

## Supplementary Materials

The following are available online at http://www.mdpi.com/2072-6643/9/10/1123/s1, Table S1: Histopathological examination results of diabetic rats.
