# Supplementary material for: Chinese Olive (Canarium album L.) Fruit Extract Attenuates Metabolic Dysfunction in Diabetic Rats
Source: Nutrients. 2017 Oct 15;9(10):1123. doi: 10.3390/nu9101123 (PMC5691739; doi:10.3390/nu9101123)
Supplement: Supplementary file 1 [file nutrients-09-01123-s001.docx]

**Table S1.** Histopathological examination results of diabetic rats.

|  |  | **Rats with results, %** | | | |
| --- | --- | --- | --- | --- | --- |
| **Item and definition** | **Score** | **Control** | **DC** | **DC+CO-EtOAc**  **(50 mg/kg)** | **DC+CO-EtOAc**  **(150 mg/kg)** |
| Steatosis grade |  |  |  |  |  |
| ＜5% | 0 | 87.5 | 0.0 | 0.0 | 0.0 |
| 5-33% | 1 | 12.5 | 0.0 | 20.0 | 40.0 |
| ＞33-66% | 2 | 0.0 | 0.0 | 20.0 | 40.0 |
| ＞66% | 3 | 0.0 | 100.0 | 60.0 | 20.0 |
| *P* value |  |  |  |  |  |
| vs. control |  |  | ＜0.05 | ＜0.05 | ＜0.05 |
| vs. DC |  |  |  | ＞0.05 | ＜0.05 |
| Lobular inflammation (foci/high-powered field  at 200X magnification) |  |  |  |  |  |
| 0 | 0 | 25.0 | 0.0 | 0.0 | 0.0 |
| ＜2 | 1 | 75.0 | 10.0 | 20.0 | 40.0 |
| 2-4 | 2 | 0.0 | 20.0 | 40.0 | 40.0 |
| ＞4 | 3 | 0.0 | 70.0 | 40.0 | 20.0 |
| *P* value |  |  |  |  |  |
| vs. control |  |  | ＜0.05 | ＜0.05 | ＜0.05 |
| vs. DC |  |  |  | ＜0.05 | ＜0.05 |
| Portal inflammation |  |  |  |  |  |
| None | 0 | 62.5 | 0.0 | 0.0 | 0.0 |
| Mild | 1 | 37.5 | 10.0 | 30.0 | 40.0 |
| Moderate | 2 |  | 30.0 | 40.0 | 40.0 |
| Severe | 3 |  | 60.0 | 30.0 | 20.0 |
| *P* value |  |  |  |  |  |
| vs. control |  |  | ＜0.05 | ＜0.05 | ＜0.05 |
| vs. DC |  |  |  | ＜0.05 | ＜0.05 |
| Hepatocellular ballooning |  |  |  |  |  |
| None | 0 | 100.0 | 0.0 | 0.0 | 0.0 |
| Few balloon cells | 1 | 0.0 | 10.0 | 30.0 | 80.0 |
| Prominent ballooning | 2 | 0.0 | 90.0 | 70.0 | 20.0 |
| *P* value |  |  |  |  |  |
| vs. control |  |  | ＜0.05 | ＜0.05 | ＜0.05 |
| vs. DC |  |  |  | ＞0.05 | ＜0.05 |
| NAS |  |  |  |  |  |
| No steatohepatitis | 0-2 | 62.5 | 0.0 | 0.0 | 0.0 |
| Borderline | 3-4 | 37.5 | 20.0 | 40.0 | 70.0 |
| Definite steatohepatitis | ≧5 |  | 80.0 | 60.0 | 30.0 |
| NAS mean value ^2^ |  | 1.9 | 5.6 | 4.2 | 3.6 |
| *P* value |  |  |  |  |  |
| vs. control |  |  | ＜0.05 | ＜0.05 | ＜0.05 |
| vs. DC |  |  |  | ＜0.05 | ＜0.05 |

^1^ Values are percentages (n = 8-10) unless otherwise stated. P values were determined using the Mann-Whitney test, evaluated by the Monte Carlo method for small samples. DC, diabetic control; CO-EtOAc, ethyl acetate fraction of Chinese olive; NAS, Non-alcoholic fatty liver disease Activity Score.

^2^ NAS score is defined as the unweighted sum of the scores for steatosis (0-3), lobular inflammation (0-3), and hepatocellular ballooning (0-2).
